# A Rose by Any Other Name: Plant Identification Knowledge & Socio-Demographics

**DOI:** 10.1371/journal.pone.0156572

**Published:** 2016-05-26

**Authors:** Beth S. Robinson, Richard Inger, Kevin J. Gaston

**Affiliations:** Environment and Sustainability Institute, University of Exeter, Penryn, Cornwall TR10 9FE, United Kingdom; Auburn University, UNITED STATES

## Abstract

Concern has been expressed over societal losses of plant species identification skills. These losses have potential implications for engagement with conservation issues, gaining human wellbeing benefits from biodiversity (such as those resulting from nature-based recreational activities), and early warning of the spread of problematic species. However, understanding of the prevailing level of species identification skills, and of its key drivers, remains poor. Here, we explore socio-demographic factors influencing plant identification knowledge and ability to classify plants as native or non-native, employing a novel method of using real physical plants, rather than photographs or illustrations. We conducted face-to-face surveys at three different sites chosen to capture respondents with a range of socio-demographic circumstances, in Cornwall, UK. We found that survey participants correctly identified c.60% of common plant species, were significantly worse at naming non-native than native plants, and that less than 20% of people recognised Japanese knotweed *Fallopia japonica*, which is a widespread high profile invasive non-native in the study region. Success at naming plants was higher if participants were female, a member of at least one environmental, conservation or gardening organisation, in an older age group (than the base category of 18–29 years), or a resident (rather than visitor) of the study area. Understanding patterns of variation in plant identification knowledge can inform the development of education and engagement strategies, for example, by targeting sectors of society where knowledge is lowest. Furthermore, greater understanding of general levels of identification of problematic invasive non-native plants can guide awareness and education campaigns to mitigate their impacts.

## Introduction

People are losing familiarity with the natural world, particularly in western countries, potentially resulting in a loss of ecological knowledge [1], including the ability to identify even the most common species, as well as those of cultural significance [1–3]. This loss of familiarity and knowledge is cause for profound concern as it may lead to reduced appreciation of the natural world [2,4,5], reduced motivation to protect species [4], less willingness to support nature conservation organisations [4], and perhaps a reduced ability to gain associated human wellbeing benefits, such as those resulting from nature-based recreational activities (e.g. bird watching; [6]). Furthermore, poor identification skills may contribute to a reduced ability or willingness to engage in documenting and monitoring biodiversity. This includes the tracking of the spread of problematic invasive non-native species, where early identification can facilitate more successful and cost effective management actions [6,7,8].

A handful of studies have examined societal knowledge of species identification, finding mixed results. For example, one investigation found Slovakian elementary school children (aged 10–15) and university students were able to identify 30–48% of common bird species [9], another that children aged 4–12 in Scotland, UK were able to identify 56, 43 and 44% of arthropod, bird and mammal species respectively (out of 40 species randomly drawn from 68; [10]), and a third that children were better able to identify artificial Pokémon characters than common native wildlife [11]. However, empirical evaluations of people’s identification skills are scarce and this is particularly true for plant species. Despite having the advantage of being immobile, relatively well described and provisioned with field guides (at least in most Western countries), plants have the disadvantage of lacking the charisma of many bird and mammal species, are significantly more diverse, are often morphologically different between seasons and life-stages, and are widely regarded as difficult to identify [3,12,13]. Those studies that have been conducted have typically found low levels of plant identification skills [3,6,14–16]. For example, visitors to urban greenspaces in the UK could on average correctly identify only one out of four plant species common to that area [6], and only 10% of 18–24 years olds in the UK could correctly identify ash *Fraxinus excelsior*, one of the most common tree species in that region [16]. This said, some studies have found higher levels of identification of common plant species, for instance 70% of participants in one analysis correctly identified buttercup *Ranunculus spp*. [1]. Studies exploring identification skills of invasive non-native species are even scarcer than those of natives. A study of the Australian public found a 20.5% error rate when distinguishing native frogs from the harmful invasive non-native cane toad *Bufo marinus* [17]. To our knowledge, however, no study has examined people’s ability to identify problematic invasive non-native plants.

Studies examining identification skills have tended to focus on particular sectors of society (e.g. students; [14]), plants associated with particular locations (e.g. [6]) and, with one notable exception [3], most have used photographs or illustrations of species. Although several socio-demographic variables have been identified as important in predicting plant identification skills, including age [1,14,16], gender [12,15] and level of education [3], their relative importance has seldom been explored. Greater understanding of socio-demographic factors influencing plant identification skills could assist with targeting awareness and educational campaigns in sections of society where knowledge is low.

In this study we ask three questions. First, which socio-demographic factors influence people’s ability to name common plants and their ability to classify plants as native or non-native? Second, how is plant identification knowledge obtained? Third, what are levels of support and motivation for learning plant identification skills? To achieve this we surveyed people with a range of socio-demographic circumstances and used real plant specimens to test identification skills.

## Methods

### Ethics statement

Permission for this survey was granted from the University of Exeter Ethics Committee. All participants were provided with a brief description of the study and gave written consent before beginning the survey. All responses were anonymous.

### Survey design

The surveys were conducted at three sites in the town of Falmouth, Cornwall, UK, during August 2013. These were chosen to capture a cross-section of society and comprised two beachside locations and one town centre location, with the goal of engaging as a wide a range of people (over 18 years of age, and UK residents) as possible. Each site was visited an equal number of times and participants were selected at random. The surveys (total n = 220) were delivered face-to-face, with one participant at a time, and were completed on site. For consistency, it was delivered by the same individual (first author, BR) in all cases. The survey comprised 14 questions (S1 Table), and was piloted several times before being formally administered to refine the method and wording of the questions, following guidance from Bernard (2011) [18].

First, participants were asked to identify samples of real plants, using a mix of fresh cuttings and potted plants purchased from a local garden centre. Using real plants, rather than images, allows the participants to gain a better idea of smell, size and texture of the plants. The plants used comprised six natives: Lavender (*Lavandula angustifolia)*, Rose spp. (Genus: *Rosa*), Common Heather (*Calluna vulgaris*), Blackberry (*Rubus fructicosus*), Ivy (*Hedera helix*) and Bracken (*Pteridium aquilinum*); and six non-natives: Hydrangea spp. (Genus: *Hydrangea*), Fuchsia spp. (Genus: *Fuchsia*), Montbretia (Genus: *Crocosmia*), Red valerian (*Centranthus ruber*), Buddleia (*Buddleja davidii*), and Japanese knotweed (*Fallopia japonica)*. This set of species was determined following consultation with experts with specialist knowledge on gardening and ecology (n = 8); these included ecological consultants, academics and garden centre employees. Each expert was asked to provide a list of 12 plants, six native and six non-native, that were relatively easy to identify, common in UK domestic gardens, and medium sized. Plants were considered non-native if they first occurred in Britain after AD 1500 [19]. Experts were asked to include native and non-native plants that are actively planted and frequently valued, as well as native and non-native plants that grow wild in gardens without assistance, and plants sometimes considered a nuisance (although this is subjective and some plants fit both criteria). The authors combined these lists with literature—both academic and non-academic—on common UK plants and their flowering times (e.g. [20] and [21]) to select the final 12 plants. The majority of plants were flowering at the time the survey was conducted. Japanese knotweed was chosen as an example of a problematic invasive non-native plant as it is considered one of the most ecologically and economically damaging invasive non-native plants in the UK, where it is widespread in a variety of habitats [22,23]. In 2010 Japanese knotweed was estimated to have cost the UK economy £165 million [24]. After giving a broad definition of ‘non-native species’ and revealing the plant names, participants were asked whether the plants (or close relatives of) were native or non-native.

The second section of the survey presented participants with statements regarding their attitudes towards plant identification, such as whether the individual thought it was an important skill to have, as well as if and how they learned their plant identification skills, and whether or not they were motivated to learn more. These questions were assessed using a five point Likert scale of ‘strongly disagree’, ‘disagree’, ‘neutral’, ‘agree’, or ‘strongly agree’ [18]. The second section also included questions addressing how much participants had been taught plant identification skills in the past and how these skills were obtained. The final section obtained data on socio-demographics (age category; gender; education level; membership of environmental, conservation or gardening organisations; garden ownership; and if participants were resident in Cornwall or elsewhere in the UK). The socio-demographic variables were chosen based on factors found to be important in explaining ecological knowledge from case studies within the academic literature (e.g. [1,3,10,14,15,16]).

The sample comprised a higher percentage of women (58.2%, n = 128) than national and Cornwall averages (50.8% and 51.6% respectively; [25], Table 1). It comprised a similar percentage in the 18–29 age category as national and Cornwall averages (17.3%, 20.6% and 20.7% respectively); a smaller percentage in the 30–39 age category (8.6%, 16.8% and 16.9% respectively); a larger percentage in both the 40–49 age category (25%, 18.6% and 18.6% respectively) and the 50–59 age category (22.7%, 15.4%, 15.4% respectively); and a similar percentage in the 60+ age category (26.3%, 28.6% and 28.5% respectively [25]).

**Table 1:** Summary statistics for socio-demographic attributes of survey participants. The shorthand used in the model outputs is followed in brackets where applicable.

**Variable**	**Summary statistics**	
**Age**		
**18–29**	17.3%	(n = 38)
**30–39**	8.6%	(n = 19)
**40–49**	25%	(n = 55)
**50–59**	22.7%	(n = 50)
**60 +**	26.3%	(n = 58)
**Gender**		
**Female**	58.2%	(n = 128)
**Male**	41.8%	(n = 92)
**Highest level of education (Education)**		
**1: ‘O’ level, GCSE, or equivalent or less**	19.6%	(n = 43)
**2: ‘A’ Level, AS Level, or equivalent**	11.4%	(n = 25)
**3: Further education or vocational training**	15.45%	(n = 36)
**4: First degree (e.g. BSc, BA)**	30%	(n = 66)
**5: Higher degree (e.g. MSc, MA, PhD)**	22.7%	(n = 50)
**If the participant was a member of a wildlife, conservation or gardening organisations (Member of)**		
**None**	49.5%	(n = 109)
**One**	25.9%	(n = 57)
**Two**	10.9%	(n = 24)
**Three or more**	13.6%	(n = 30)
**If the participant had a garden (Garden)**		
**Yes**	90%	(n = 198)
**No**	10%	(n = 22)
**Where the participant currently lives (Lives)**		
**Cornwall**	48.1%	(n = 108)
**Rest of UK**	50.9%	(n = 112)

### Statistical analysis

All analyses were carried out in R (3.0.3) [26]. No collinearity was found between explanatory variables (assessed using Pearson’s correlation coefficient with cut-off <0.8) [27]. Generalised linear mixed effect models (using the ‘lme4’ package [28]) with a binomial error structure were constructed to explore the effect of socio-demographic factors selected *a priori* (age; gender; education; membership of environmental, conservation or gardening organisations; garden ownership; and if participants were resident in Cornwall or elsewhere in the UK–all categorical) on participants’ abilities to name plants and classify plants as native or non-native (response variables). The response variables were entered into the model as number of correct answers minus the number of incorrect answers (using the ‘cbind’ function). Survey location was included as a random factor.

The global model contained all explanatory variables chosen *a priori*. Simplification of the global model was achieved based on Akaike Information Criterion (AIC) using the ‘MuMIn’ package [29] which holds functions to compare all possible sub-sets of the global model. All models with ΔAIC <6 were retained [30,31]. Model averaging was used to calculate averaged parameter estimates and assess the relative importance of parameters using the natural averaging method [32]. R^2^ values were calculated for the global models using the method described by Nakagawa and Schielzeth [33]. Parameters within the resulting averaged model were considered significant if the p-value was <0.05.

To test whether participants were better at identifying native or non-native plants a Mann Whitney U test was carried out as the data were not normally distributed. To explore if there was a correlation between participants’ abilities to name plant species and their abilities to classify them as native or non-native, a Kendall’s rank correlation test was carried out, as again the data were not normally distributed.

The relationships between responses to Likert-style questions exploring levels of support and motivation for learning plant identification skills (questions 3 to 6; ordinal data) and socio-demographic factors were analysed using a cumulative link model using the “ordinal” package [34]. Model averaging followed this using the method outlined above. Models using all five response categories did not converge, therefore these were condensed to three categories–‘agree’ (agree and strongly agree), ‘neutral’ and ‘disagree’ (disagree and strongly disagree).

## Results

**Fig 1:**
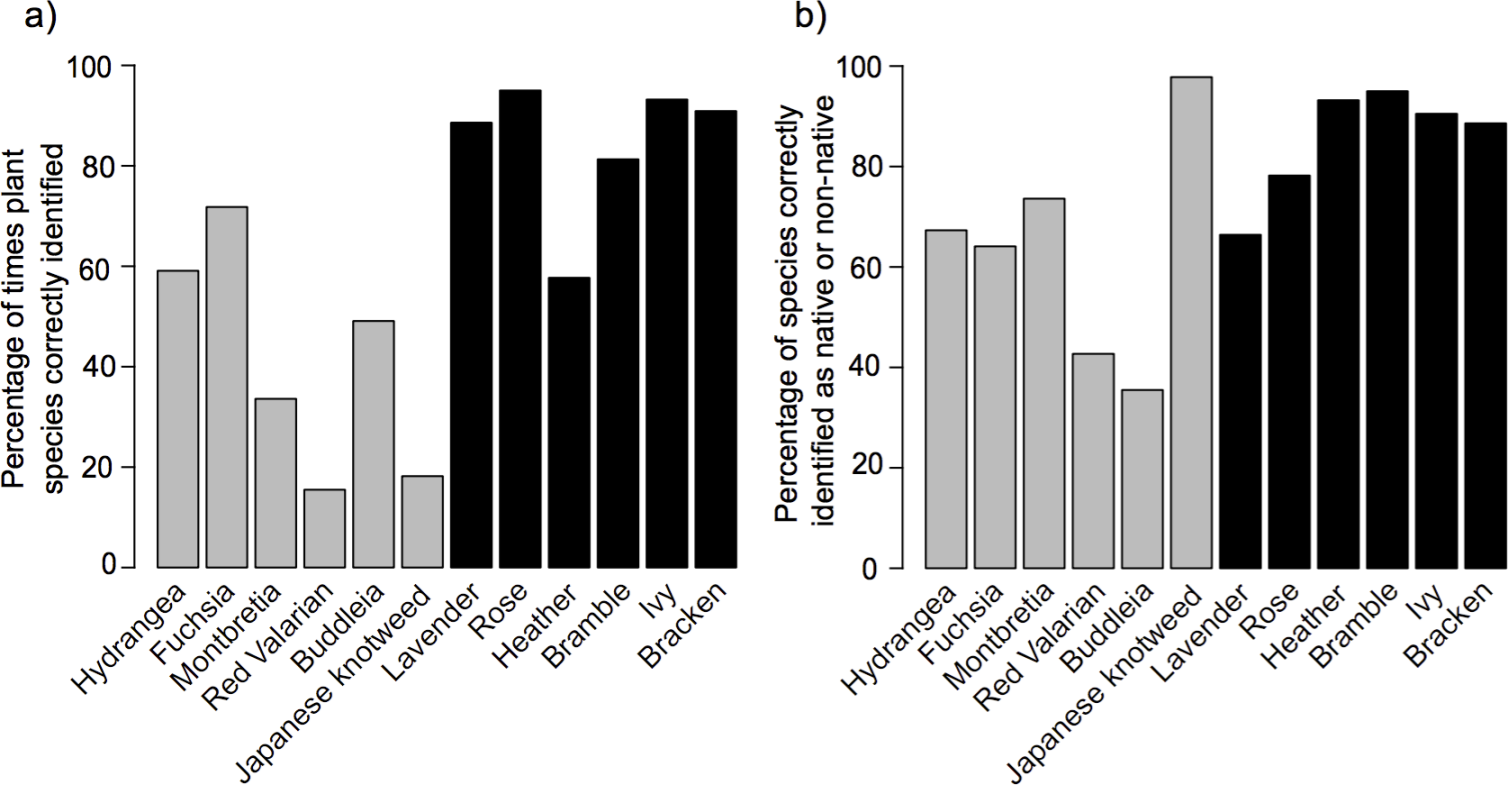
**Results of plant identification survey for a) percentage of times each plant was correctly identified; b) percentage of times each plant was correctly classified as native or non-native**. Light grey bars = non-native species; black bars = native species.

Participants scored a mean of 62.8% (s.e. = 0.19) when naming plant species (see S2 Table for full survey results). Participants were better at identifying plants if they were older, a member of an environmental, conservation or gardening organisation, if they were female, and if they lived in Cornwall (Tables 2A and 3A; see S3 Table for results of global model). The model explained 14% of variation in the ability to identify plant species (marginal R^2^ = 0.138; conditional R^2^ = 0.141). Participants were significantly better at identifying native than non-native plants (Mann-Whitney U test, W = 2, p = 0.009). Japanese knotweed and Red valerian were correctly identified by less than 20% of participants (Fig 1A).

**Table 2:** Summary of results after model averaging for a) ability to name plants and b) ability to classify plants as native or non-native. See Table 1 for descriptions of explanatory variables. The base categories were: female; education level 1 (‘O’ level, GCSE, or equivalent or less); member of no wildlife, conservation or gardening organisations; if the participant did not a have a garden; and if the participant was currently a resident in Cornwall.

	Parameter Estimate	Standard Error	Adjusted Standard Error	z-value	P-value
a) Ability to correctly name plants					
Intercept	-0.276	0.180	0.181	1.529	0.126
Age (30–39)	0.572	0.174	0.175	3.263	0.001
Age (40–49)	0.870	0.135	0.136	6.394	< 0.001
Age (50–59)	1.179	0.142	0.142	8.288	< 0.001
Age (60+)	1.397	0.147	0.147	9.477	< 0.001
Gender (male)	-0.699	0.089	0.089	7.819	< 0.001
Member of (one)	0.308	0.112	0.113	2.739	0.006
Member of (two)	0.424	0.140	0.141	3.005	0.003
Member of (three)	0.682	0.149	0.150	4.547	< 0.001
Garden (yes)	0.259	0.149	0.150	1.724	0.085
Lives (rest of UK)	-0.232	0.095	0.096	2.424	0.015
b) Ability to classify plants as native or non-native					
Intercept	0.875	0.168	0.169	5.180	< 0.001
Age (30–39)	0.162	0.188	0.189	0.857	0.391
Age (40–49)	0.168	0.139	0.140	1.203	0.229
Age (50–59)	0.191	0.142	0.143	1.339	0.181
Age (60+)	0.015	0.135	0.136	0.111	0.912
Gender (male)	0.189	0.092	0.093	2.041	0.041
Education (2)	0.137	0.159	0.160	0.853	0.394
Education (3)	0.112	0.150	0.150	0.745	0.456
Education (4)	0.197	0.129	0.130	1.512	0.130
Education (5)	0.343	0.140	0.141	2.438	0.015
Member of (one)	-0.060	0.109	0.109	0.552	0.581
Member of (two)	0.104	0.148	0.149	0.697	0.486
Member of (three)	0.151	0.139	0.140	1.082	0.279
Garden (yes)	0.194	0.148	0.149	1.304	0.192
Lives (rest of UK)	-0.009	0.092	0.092	0.097	0.923

**Table 3:** Results of top 10 models based on AIC_c_. df = degrees of freedom, weight = Akaike weight. See Table 1 for detailed descriptions of explanatory variables.

Intercept		df	Log-likelihood	AICc	ΔAICc	weight
a) Ability to correctly name plants						
-0.332	Age + Gender + Member of + Garden + Live	12	-479.07	983.6	0.00	0.514
-0.163	Age + Gender + Member of + Live	11	-480.65	984.6	0.93	0.323
-0.268	Age + Gender + Member of	10	-483.36	987.8	4.14	0.065
-0.412	Age + Gender + Member of + Garden	11	-482.32	987.9	4.27	0.061
-0.288	Age + Gender + Education + Member of + Garden + Live	16	-477.65	990.0	6.34	0.022
-0.097	Age + Gender + Education + Member of + Live	15	-479.41	991.2	7.53	0.012
-0.209	Age + Gender + Education + Member of	14	-482.32	994.7	11.06	0.002
-0.368	Age + Gender + Education + Member of + Garden	15	-481.21	994.8	11.14	0.002
-0.200	Age + Gender + Garden + Live	9	-492.45	1003.8	20.13	0.000
0.019	Age + Gender + Live	8	-494.89	1006.5	22.83	0.000
b) Ability to classify plants as native or non-native						
0.990	Gender	3	-407.631	821.4	0.000	0.188
0.819	Gender + Garden	4	-406.820	821.8	0.450	0.150
0.993	Gender + Live	4	-407.629	823.4	2.070	0.067
0.614	Gender + Garden + Education	8	-403.476	823.6	2.260	0.061
0.837	Gender + Education	7	-404.657	823.8	2.470	0.055
0.824	Gender + Garden + Live	5	-406.800	823.9	2.510	0.054
0.924	Garden	3	-409.154	824.4	3.050	0.041
0.968	Gender + Member of	6	-406.360	825.1	3.740	0.029
0.897	Education	6	-406.418	825.2	3.860	0.027
0.702	Garden + Education	7	-405.477	825.5	4.110	0.024

Participants scored a mean of 74.4% (s.e. = 0.11) when classifying plants as native or non-native, with Buddleia most commonly misclassified (Fig 1B). Participants better at classifying plants as native or non-native were male and had post-graduate qualifications (Tables 2B and 3B; see S3 Table for results of global model). However, the model explained approximately 1% of variation in the ability to distinguish native/non-native plants (marginal and conditional R^2^ = 0.010). There was no correlation between participants’ abilities to name plant species and their ability to classify them as native or non-native (Kendall’s rank correlation, z = 1.25, p = 0.21).

**Fig 2:**
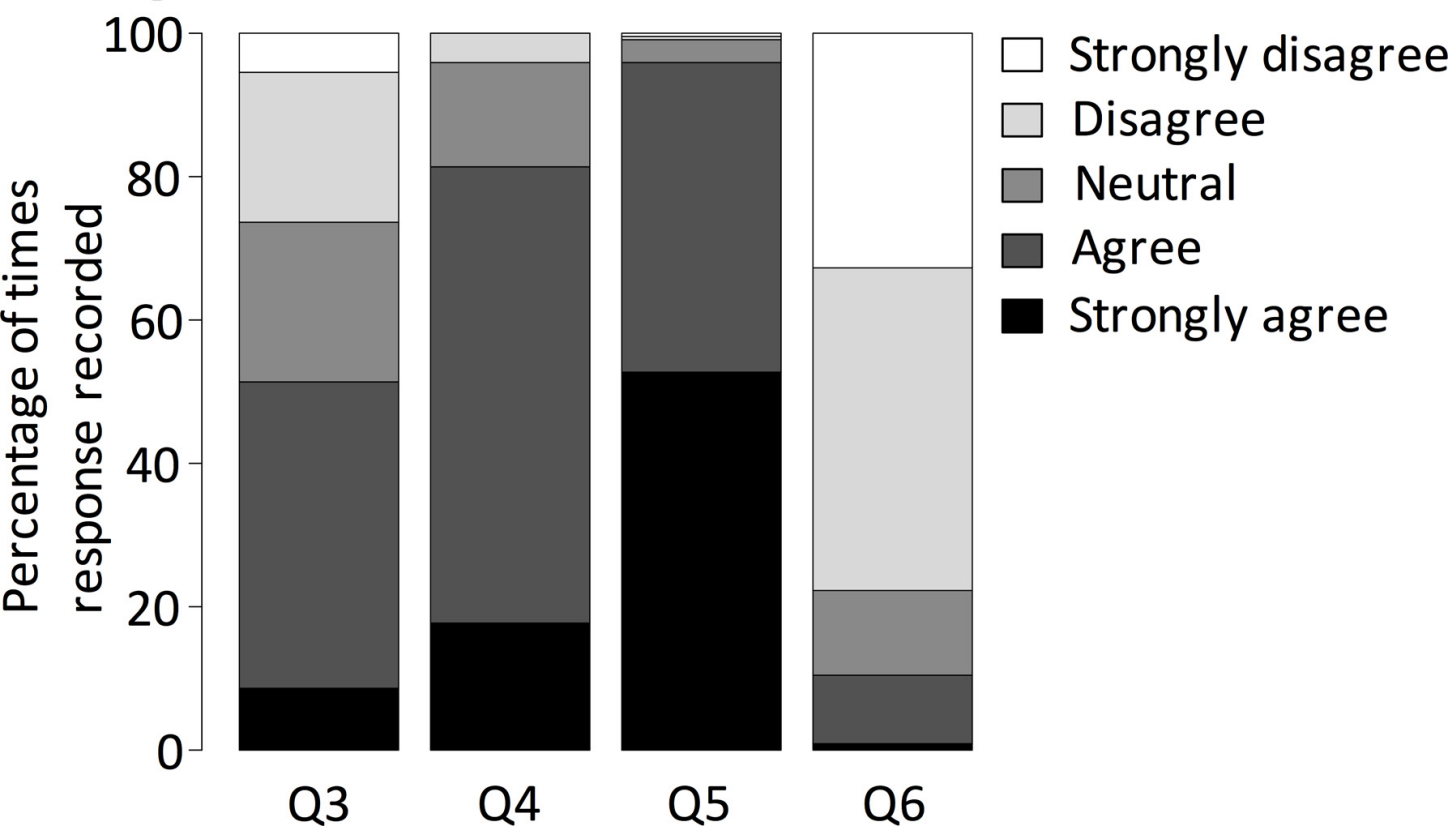
Responses to Likert-Style questions about attitudes towards plant identification and motivation to learn. Survey responses for Q3: Knowing the names of plants is important to me; Q4: I think children should be taught how to identify common plant species; Q5: If given the opportunity to improve my plant identification knowledge I would take it; and Q6: I have no motivation to learn the names of plants.

About half of participants (51.8%, n = 114) agreed or strongly agreed that knowing the names of plants was important to them. Higher levels of support were reported for children being taught plant names, for taking opportunities to learn plant names, and the majority (80%, n = 176) disagreed or strongly disagreed that they had no motivation to learn plant names (Fig 2). Socio-demographic factors were not significantly related to any responses, with the exception of gender for question 6—‘I have no motivation to learn the names of plants’ (p = 0.009, confidence intervals = 0.24, 1.67, estimate = 0.95, standard error = 0.36, z value = 2.61). 26.8% (n = 59) of participants reported being taught plant identification skills ‘a lot’, 34.5% (n = 76) ‘some’, 31.8% (n = 70) ‘a little’ and 6.8% (n = 15) ‘never’. Of the last group, five participants reported no methods of being taught and 10 reported being self-taught. 80% (n = 176) of participants reported learning plant identification skills from family, 47.7% (n = 105) being self-taught, 31.8% (n = 70) learnt at school, and 8.6% (n = 19) by attending courses.

## Discussion

Using a novel methodological approach with real plants, rather than photographs or illustrations, this study asked three questions: (1) which socio-demographic factors influence people’s ability to name common plants and their ability to classify plants as native or non-native? (2) how is plant identification knowledge obtained?, and (3) what are levels of support and motivation for learning plant identification skills? The results of our study suggest that participants from a broad cross-section of society were better at correctly naming plants if they were female, a member of at least one environmental, conservation or gardening organisation, in an older age group, or a resident (rather than a visitor) in the study area. Conversely, success at identifying plants as native or non-native was higher if participants were male and had post-graduate qualifications. Overall participants correctly identified c.60% of common plant species, and were poor at recognising Japanese knotweed (*Fallopia japonica*), which is a widespread high profile invasive non-native in the study region. Here we discuss our findings and their implications for engagement with conservation issues, the potential to gain human wellbeing benefits from biodiversity, and to engage in monitoring and tracking of biodiversity, including early warning of problematic species.

Participants in this study scored significantly lower when naming non-native compared with native plants–this is similar to a previous study that found children were worse at identifying non-native than native arthropods, birds and mammals [10]. Although not all non-native plants cause damage, those that do can have significant ecological and socio-economic costs [8]. Of particular concern is that the widespread and problematic invasive non-native plant Japanese knotweed [23] was the second least identifiable plant. Japanese knotweed causes widespread ecological damage and can be expensive to control [22,35]. In the UK Japanese knotweed is found within domestic gardens, where some of the problems it causes, particularly economic ones, can be most acute [36]. Given the private nature of domestic gardens, and the fact that many back gardens in particular, are secluded from view by passers-by, the presence of invasive non-native plants will be observed by only a few. It is therefore important that identification skills of such plants are high amongst the public to increase the chances of early identification and therefore successful and cost effective eradication.

The ability to identify invasive non-native plants in both domestic gardens and in the wider landscape is also important because it allows people to contribute towards citizen science projects that track them [37]. Data generated this way are valuable for scientific research exploring the drivers of the distribution of invasive non-native plants [38,39], which subsequently can inform policy and management recommendations to reduce their ecological and socio-economic impacts [40].

Participants were surprisingly good at identifying common native plants compared with most previous research using traditional methods of pictures and illustrations. For example, one study in Sheffield, UK found that visitors to an urban green space could identify only one out of four plant species common to that area from photographs [6]. Another found that 86% of UK A-level students could only identify three or less out of ten wild flowers from illustrations [14]. This raises the possibility that the way in which identification skills are tested could be particularly important to the outcome. Using real plants is more reminiscent of the way people recognise and engage with plants in the environment as participants can smell and touch them, and gain a better idea of their size. Stagg and Donkin (2013) [3] also used real plants to test identification skills, however they used potted weeds, fresh winter twigs and dried seed heads. The latter two are less representative of what one would see in the environment, and may explain why some participants in that study had poor plant identification skills.

Half of survey participants were members of at least one environmental, conservation or gardening organisation, which was a significant factor in predicting better identification skills. Although comparable data are scarce, this may be higher than in other settings (e.g. 26% of participants in a survey in Scotland were members of ‘wildlife, conservation or heritage’ organizations; [41]). It is likely that people who are more interested in nature to begin with are more likely to join such organisations, and therefore reap the benefits such membership provides, such as increased access to nature reserves, volunteer opportunities and regularly receiving magazines filled with nature-related content. However, there is likely to be some level of positive feedback once joined, and that membership of such organisations encourages greater interest in and engagement with nature, thus leading to better plant identification skills. Membership of environmental and conservation organisations has been shown to be correlated with other types of nature-related knowledge (e.g. status of protected areas in the UK by their users [42]), and environmental behaviour (e.g. willingness to pay to prevent oil pollution in coastal areas [43]).

Our survey also asked about membership of gardening organisations, responses to which included national organisations such as the Royal Horticultural Society and local gardening organisations. The reasons for joining, and benefits gained from, gardening organisations will likely differ from those for conservation and environmental organisations. Nevertheless, membership of such organisations could likely be an important way for people to improve plant identification skills. Increasing membership of conservation and environmental, as well as gardening organisations, and working through and with them is potentially an important tool for engaging the public with nature [42], and thus improving plant identification skills.

Participants from Cornwall were significantly better at naming plants than participants from the rest of the UK. This could be explained by the much higher percentage of people in Cornwall who live in rural areas compared with the rest of the UK (61.4% and 18.5% respectively; [25]). The increasing percentage of the world’s population living in urbanized areas (predicted to be >80% by 2050 [44]) has frequently been linked to reduced access and opportunities to engage with nature [2,4,5], which could lead to loss of plant identification skills. Promoting opportunities for urban residents to access green spaces could help mitigate this trend [6,45].

Age was the strongest predictor of plant identification knowledge, with those aged 60 and over being the best at naming plants. This correlation is consistent with previous research (e.g. [14,16]). Age has also been found to be an important factor in other ways that people engage with nature, for example, participation in bird feeding activities [46]. It is, however, difficult to determine the causality of the relationship and whether younger generations will gain plant identification skills later in life or if this knowledge is being lost in younger generations. As evidence indicates that encounters with nature at an early age are important for promoting connections with nature [5], encouraging nature experiences for younger generations should be a priority if we are to increase motivation to learn and improve plant identification skills.

To improve plant identification knowledge, pathways by which people commonly obtain such knowledge could be invested in and promoted. In this study these were via family and by being self-taught, which might be considered ‘less formal’ methods of learning. The least frequently reported pathways of learning plant identification skills were through more formal methods—at school and through attending courses. It is important to consider how investment in these less frequently reported pathways could improve plant identification knowledge. Consideration could be given to how changes to the school curriculum and creative methods of teaching used can rectify this, as called for by others [3,10,12]. Attending courses was the least frequent way participants reported learning plant identification skills: only 8.6% of participants reported learning this way. Whilst attending courses of this type is not suited to everyone’s taste, consideration of potential barriers to attending such courses, such as time and money–although these might also be deeper rooted and due to cultural and social differences—could assist in unlocking the potential in learning via this method.

To implement practical actions to improve species identification skills people first need to be aware of the importance of identification skills and have motivation to improve them. However, the contrast between only half of participants agreeing or strongly agreeing that learning plant names is important, and the high support reported for taking opportunities to improve plant identification skills, further highlights that the relationship and gap between ecological attitudes and ecological behavior is complex and difficult to address [47,48]. Therefore, any measures to improve plant identification skills need to address the reasons why people learn plant identification skills and why some do not think they are important; more qualitative research techniques, perhaps using in-depth interviews could assist with this.

There were several limitations to this study. The factors in the model explaining plant identification, and even more so, the model exploring knowledge of what was native or non-native explained little of the variation. However, the overall conclusions were not changed by the model selection process as the same factors were significant in the global models (S3 Table). This low explanatory power suggests that there are other unexplored variables contributing to these types of knowledge. It is also important to remember that this study was carried out in a ‘post-industrial nation’, where despite some plants still having cultural significance, plant identification skills have often become irrelevant for daily needs. For example, one study found that plant identification knowledge was much higher in India and Indonesia than in the UK, which they attribute to differences in culture and resource dependence in the three countries [1]. Therefore, perhaps the challenge lies in establishing the relevance and worth of plant identification skills [1]. One way in which this might already be happening for plants that have practical uses is through the renewed interest in foraging wild foods, particularly by younger generations [49,50]. Blackberries or bramble *Rubus fructicosus*, are the best example from our results to demonstrate this as they are one of the most commonly foraged foods in the UK [50].

The biggest challenge is perhaps how to increase motivation to learn identification skills for plants that do not have practical uses or cultural significance, such as some of the non-native species, particularly problematic invasive ones. To address this challenge there needs to be an increase in societal awareness and understanding of the relative costs and benefits of different non-native plants to biodiversity, as well as an increased awareness of the importance of early identification and eradication of problematic non-native plants. It is of profound importance that the challenges addressed in this study continue to be addressed to increase the likelihood that the benefits plant identification skills can bring are delivered, such as increased engagement with conservation issues, potential human wellbeing benefits, the monitoring of problematic species, and increased connectedness with nature [2,4,6].

## Supporting Information

S1 Table Outline of plant identification survey. (DOCX)

S2 Table Complete results of plant identification survey. (XLS)

S3 Table Results of global model. (DOCX)

## References

[1] Pilgrim SE, Cullen LC, Smith DJ, Pretty J. Ecological knowledge is lost in wealthier communities and countries. Environ Sci Technol. 2008;42: 1004–1009. 10.1021/es070837v 18351064

[2] Miller JR. Biodiversity conservation and the extinction of experience. Trends Ecol Evol. 2005;20: 430–4. 10.1016/j.tree.2005.05.013 16701413

[3] Stagg BC, Donkin M. Teaching botanical identification to adults: experiences of the UK participatory science project “Open Air Laboratories”. J Biol Educ. 2013;47: 104–110. 10.1080/00219266.2013.764341

[4] Pyle RM. Nature matrix: reconnecting people and nature. Oryx. 2003;37: 206–214. 10.1017/S0030605303000383

[5] Stokes DL. Conservators of Experience. Bioscience. 2006;56: 6–7. 10.1641/0006-3568(2006)056[0007:COE]2.0.CO;2

[6] Dallimer M, Irvine KN, Skinner AMJ, Davies ZG, Rouquette JR, Maltby LL, et al Biodiversity and the feel-good factor: understanding associations between self-reported human well-being and species richness. Bioscience. 2012;62: 47–55. 10.1525/bio.2012.62.1.9

[7] Dermott SM, Irwin RE, Taylor BW. Using economic instruments to develop effective management of invasive species: insights from a bioeconomic model. Ecol Appl 2013;23: 1086–1100. 10.1890/12-0649.1 23967577

[8] Simberloff D, Martin JL, Genovesi P, Maris V, Wardle DA, Aronson J, et al Impacts of biological invasions: what’s what and the way forward. Trends Ecol Evol. 2013;28: 58–66. 10.1016/j.tree.2012.07.013 22889499

[9] Prokop P, Rodak R. Ability of Slovakian pupils to identify birds. Eurasia J. Math. Sci. 2009;5: 127–133.

[10] Huxham M, Welsh A, Berry A, Templeton S. Factors influencing primary school children’s knowledge of wildlife. J. Biol. Educ. 2006;41: 9–13. 10.1080/00219266.2006.9656050

[11] Balmford A, Clegg L, Coulson T, Taylor J, Street D. Why conservationists should heed Pokémon. Sci. Mag. 2002;295: 5–6. 10.1126/science.295.5564.2367b 11924673

[12] Wandersee JH, Schussler EE. Preventing plant blindness. Am Biol Teach. 1999;61: 82–86. 10.2307/4450624

[13] Schussler EE, Olzak LA. It’s not easy being green: student recall of plant and animal images. J Biol Educ. 2008;42: 112–119. 10.1080/00219266.2008.9656123

[14] Bebbington A. The ability of A-level students to name plants. J. Biol. Educ. 2005;39: 62–67. 10.1080/00219266.2005.9655963

[15] Gatt S, Tunnicliffe SD, Borg K, Lautier K. Young Maltese children’s ideas about plants. J Biol Educ. 2007;41: 117–122. 10.1080/00219266.2007.9656080

[16] Woodland Trust, YouGov. YouGov / Woodland Trust Survey Results. 2013. Available: http://d25d2506sfb94s.cloudfront.net/cumulus_uploads/document/048vpv8neq/YG-Archive-Woodland-Trust-survey-results-240613-native-trees.pdf. Accessed: 19 February 2015

[17] Somaweera R, Somaweera N, Shine R. Frogs under friendly fire: How accurately can the general public recognize invasive species? Biol Conserv. 2010;143: 1477–1484. 10.1016/j.biocon.2010.03.027

[18] Bernard HR. Research Methods in Anthropology, 5^th^ edn. AltaMira, Plymouth; 2011. pp. 187–222.

[19] Maskell LC, Firbank LG, Thompson K, Bullock JM, Smart SM. Interactions between non-native plant species and the floristic composition of common habitats. J Ecol 2006;94: 1052–1060. 10.1111/j.1365-2745.2006.01172.x

[20] RHS. Plants that flower in August. Available: http://www.rhsplants.co.uk/plants/_/vid.84/start.2. Accessed: 16 July 2015.

[21] Streeter D, Hart-Davies C, Hardcastle A, Cole F, Harper L. Collins Flower Guide (Britain and Ireland). London: Harper Collins; 2010.

[22] Engler J, Abt K, Bunk C. Seed characteristics and germination limitations in the highly invasive *Fallopia japonica* s.l. (Polygonaceae). Ecol Res. 2011;26: 555–562. 10.1007/s11284-011-0813-8

[23] Gozlan RE, Burnard D, Andreou D, Britton JR. Understanding the threats posed by non-native species: public vs. conservation managers. PLoS One. 2013;8: e53200 10.1371/journal.pone.0053200 23341931 PMC3547005

[24] Williams F, Eschen R, Harris A, Djeddour D, Pratt C, Shaw RS, et al. The Economic Cost of Invasive Non-Native Species on Great Britain. 2010. Available: http://www.nonnativespecies.org/index.cfm?sectionid=59

[25] Office for National Statistics. Census 2011. London, UK: ESRC/JISC Census Programme. Available: http://www.ons.gov.uk/ons/index.html

[26] R Development Core Team. R: a language and environment for statistical computing R Foundation for Statistical Computing, Vienna, Austria 2013 Available: http://www.r-project.org. Accessed: February 2013.

[27] Dormann CF, Elith J, Bacher S, Buchmann C, Carl G, Carr G, et al Collinearity: a review of methods to deal with it and a simulation study evaluating their performance. Ecography. 2013;36: 27–46. 10.1111/j.1600-0587.2012.07348.x

[28] Bates D, Maechle M, Bolker B, Walker S. lme4: Linear mixed-effects models using Eigen and S4. Version 1.1–7. 2014. Available: http://cran.r-project.org/web/packages/lme4/index.html.

[29] Barton K. MuMIn: Multi-model inference. Version 1.0. 2011. Available: http://cran.r-project.org/web/packages/MuMIn/index.html.

[30] Richards SA. Dealing with overdispersed count data in applied ecology. J Appl Ecol. 2008;45: 218–227. 10.1111/j.1365-2664.2007.01377.x

[31] Richards SA, Whittingham MJ, Stephens PA. Model selection and model averaging in behavioural ecology: the utility of the IT-AIC framework. Behav Ecol Sociobiol. 2010;65: 77–89. 10.1007/s00265-010-1035-8

[32] Burnham KP, Anderson DR. Model Selection and Multimodel Inference. Springer-Verlag New York, Inc 2002.

[33] Nakagawa S, Schielzeth H. A general and simple method for obtaining R2 from generalized linear mixed-effects models. Methods Ecol Evol. 2013;4: 133–142. 10.1111/j.2041-210x.2012.00261.x

[34] Christensen R. Package “ordinal”. Regression Models for Ordinal Data. 2014 Available: http://www.cran.r-project.org/web/packages/ordinal/index.html.

[35] Shaw RH. Japanese knotweed, journalism and the general public. EPPO Bulletin. 2014;44: 1–4. 10.1111/epp.12114

[36] Royal Institution of Chartered Surveyors (RICS). Japanese Knotweed and residential property. RICS information paper. 2012.

[37] Crall AW, Newman GJ, Jarnevich CS, Stohlgren TJ, Waller DM, Graham J. Improving and integrating data on invasive species collected by citizen scientists. Biol. Invasions. 2010;12: 3419–3428. 10.1007/s10530-010-9740-9

[38] Wallace RD, Bargeron CT. Identifying invasive species in real time: Early detection and distribution mapping system (EDDMapS) and other mapping tools In: Kiska LH, Dukes JS, editors. Invasive species and global climate change. Wallingford and Boston: CABI; 2014 pp. 219–230.

[39] Gallo T, Waitt D. Creating a successful citizen science model to detect and report invasive species. Bioscience. 2011;61: 459–465. 10.1525/bio.2011.61.6.8

[40] Mackechnie C, Maskell L, Roy D. The role of ‘Big Society’ in monitoring the state of the natural environment. J. Environ. Monit. 2011;13: 2687–2691. 10.1039/c1em10615e 21879098

[41] Bremner A, Park K. Public attitudes to the management of invasive non-native species in Scotland. Biol Conserv. 2007;139: 306–314. 10.1016/j.biocon.2007.07.005

[42] Booth JE, Gaston KJ, Armsworth PR. Public understanding of protected area designation. Biol Conserv. 2009;142: 3196–3200. 10.1016/j.biocon.2009.07.024

[43] Liu X, Wirtz KW, Kannen A, Kraft D. Willingness to pay among households to prevent coastal resources from polluting by oil spills: A pilot survey. Mar. Pollut. Bull. 2009;58: 1514–1521. 10.1016/j.marpolbul.2009.05.015 19539336

[44] United Nations Population Fund. UNFPA state of world population 2007: unleashing the potential of urban growth United Nations Population Fund, New York, New York, USA 2007.

[45] Lin BB, Fuller RA, Bush R, Gaston KJ, Shanahan DF. Opportunity or orientation? Who uses urban parks and why. PLoS One 2014;9: 1–7. 10.1371/journal.pone.0087422 PMC3906185 24489913

[46] Davies ZG, Fuller RA, Dallimer M, Loram A, Gaston KJ. Household factors influencing participation in bird feeding activity: a national scale analysis. PLoS One. 2012;7: 1–10. 10.1371/journal.pone.0039692 PMC3386264 22761872

[47] Clayton S, Myers G. Chapter 2: Attitudes, values and perceptions In: Conservation Psychology: Understanding and Promoting Human Care for Nature. Wiley-Blackwell, Chichester; 2009 pp.16–33.

[48] Kaiser FG, Fuhrer U. Ecological behavior’s dependency on different forms of knowledge. Appl. Psychol. 2003:52; 598–614. 10.1111/1464-0597.00153

[49] Lee J, Garikipati S. Negotiating the non-negotiable: British foraging law in theory and practice. J Environ Law. 2011;23: 415–439. 10.1093/jel/eqr014

[50] Price K, Randall N. Foraging for wild food in the UK. Harper Adams University project report 2014; 107. Available: http://www.openfields.org.uk/. Accessed 9^th^ February 2015

