# Supplementary material for: A Rose by Any Other Name: Plant Identification Knowledge & Socio-Demographics
Source: PLoS One. 2016 May 26;11(5):e0156572. doi: 10.1371/journal.pone.0156572 (PMC4881975; doi:10.1371/journal.pone.0156572)
Supplement: S1 Table — (DOCX) [file pone.0156572.s001.docx]

**Supplementary Material for ‘A rose by any other name: plant identification skills & socio-demographics’**

Beth S. Robinson, Richard Inger & Kevin J. Gaston

**Table S1:** Outline of plant identification survey.

| **Section one: Plant identification skills** |
| --- |
| Displayed in front of you are 12 plants that are frequently found in UK gardens.  Q1. Please name the plants displayed that are numbered 1 – 12  Q2. Knowing the name of the plant do you think it (or close relatives of) could be native or non-native to the UK?   \| **Plant number** \| **Name of Plant** \| **Do you think the plant (or close**  **relatives of) could be native?**  Yes / No / Don’t know \| \| --- \| --- \| --- \| \| **1** \|  \|  \| \| **2** \|  \|  \| \| **3** \|  \|  \| \| **4** \|  \|  \| \| **5** \|  \|  \| \| **6** \|  \|  \| \| **7** \|  \|  \| \| **8** \|  \|  \| \| **9** \|  \|  \| \| **10** \|  \|  \| \| **11** \|  \|  \| \| **12** \|  \|  \| |
| **Sections two: Attitudes towards plant identification skills** |
| Please read the statements carefully and *circle* the response that most accurately describes how you feel about the statement. |
| \| Q3. Knowing the names of plants is important to me. \| Strongly disagree \| Disagree \| Neutral \| Agree \| Strongly Agree \| \| \| --- \| --- \| --- \| --- \| --- \| --- \| --- \| \| Q4. If given the opportunity to improve my plant identification knowledge I would take it. \| Strongly disagree \| Disagree \| Neutral \| Agree \| Strongly Agree \| \| \| Q5. I think children should be taught how to identify common plant species. \| Strongly disagree \| Disagree \| Neutral \| Agree \| Strongly Agree \| \| \| Q6. I have no motivation to learn the names of plants. \| Strongly disagree \| Disagree \| Neutral \| Agree \| Strongly Agree \| \| \| Q7. I have been taught the names of plants in the past. \| Never \| A little \| Some \| A lot \| \| \| Q8. How were you taught? (Please *circle* all relevant)  By family members School Attending course(s) Self taught  Other………...………………….... \| \| \| \| \| \| |
| **Section three: Background information** |
| Q9. How old are you? (Please *circle*)  18 – 29 30 – 39 40 – 49 50 – 59 60+  Q10. What is your gender? (Please *circle*)  Female Male  Q11. What is your highest level of education you have completed? (Please *circle*)  No formal qualifications  ‘O’ level, GCSE, *or equivalent*  ‘A’ Level, AS Level, *or equivalent*  Further education or vocational training (e.g. BTEC, City and Guilds)  First degree (e.g. BSc, BA)  Higher degree (e.g. MSc, MA, PhD, PGCE, post-graduate certificates/diplomas)  Q12. Are you a member of any environmental, conservation or gardening organisations?   \| Yes (please select from list) No  National Trust  Local Wildlife Trust  Woodland Trust  Friends of the Earth  Royal Botanic Gardens  World Wildlife Fund  Royal Society for the Protection of Birds  Greenpeace  RHS  Other(s) (please specify) __________________________________________________________ \|  \| \| \| --- \| --- \| --- \| \| Q13. Do you have a garden? (Please *circle*)  Yes No  Q14. Where do you currently live? (Please circle) Cornwall Rest of UK \| \| |
