# Supplementary material for: A Rose by Any Other Name: Plant Identification Knowledge & Socio-Demographics
Source: PLoS One. 2016 May 26;11(5):e0156572. doi: 10.1371/journal.pone.0156572 (PMC4881975; doi:10.1371/journal.pone.0156572)
Supplement: S3 Table — (DOCX) [file pone.0156572.s003.docx]

|  |  |  |  |  |  |  |  |
| --- | --- | --- | --- | --- | --- | --- | --- |

**Table S3:** Results from global models. See Table 1 in main manuscript for descriptions of explanatory variables.

|  | Estimate | Std. Error | z value | P-value |
| --- | --- | --- | --- | --- |
| a) Ability to correctly name plants | | |  |  |
| (Intercept) | -0.288 | 0.209 | -1.378 | 0.168 |
| Age (30-39) | 0.553 | 0.176 | 3.147 | 0.002 |
| Age (40-49) | 0.837 | 0.135 | 6.222 | <0.001 |
| Age (50-59) | 1.140 | 0.142 | 8.027 | <0.001 |
| Age (60+) | 1.345 | 0.151 | 8.900 | <0.001 |
| Gender (male) | -0.679 | 0.089 | -7.649 | <0.001 |
| Education (2) | -0.196 | 0.164 | -1.197 | 0.231 |
| Education (3) | -0.113 | 0.156 | -0.725 | 0.468 |
| Education (4) | 0.002 | 0.140 | 0.014 | 0.989 |
| Education (5) | -0.002 | 0.148 | -0.014 | 0.989 |
| Member of (one) | 0.282 | 0.113 | 2.497 | 0.013 |
| Member of (two) | 0.434 | 0.141 | 3.068 | 0.002 |
| Member of (three) | 0.655 | 0.152 | 4.305 | <0.001 |
| Garden (yes) | 0.282 | 0.150 | 1.874 | 0.061 |
| Lives (rest of UK) | -0.256 | 0.096 | -2.657 | 0.008 |
| b) Ability to classify plants as native or non-native | | | |  |
| (Intercept) | 0.606 | 0.198 | 3.066 | 0.002 |
| Age (30-39) | 0.094 | 0.192 | 0.490 | 0.624 |
| Age (40-49) | 0.119 | 0.146 | 0.818 | 0.414 |
| Age (50-59) | 0.153 | 0.152 | 1.012 | 0.312 |
| Age (60+) | -0.007 | 0.157 | -0.044 | 0.965 |
| Gender (male) | 0.191 | 0.094 | 2.024 | 0.043 |
| Education (2) | 0.105 | 0.170 | 0.618 | 0.537 |
| Education (3) | 0.085 | 0.153 | 0.556 | 0.579 |
| Education (4) | 0.195 | 0.141 | 1.379 | 0.168 |
| Education (5) | 0.295 | 0.150 | 1.966 | 0.049 |
| Member of (one) | -0.063 | 0.111 | -0.564 | 0.573 |
| Member of (two) | 0.110 | 0.149 | 0.737 | 0.461 |
| Member of (three) | 0.110 | 0.148 | 0.747 | 0.455 |
| Garden (yes) | 0.206 | 0.160 | 1.288 | 0.198 |
| Lives (rest of UK) | -0.065 | 0.097 | -0.667 | 0.505 |
